# Mesenchymal stem cells attenuate inflammatory processes in the heart and lung via inhibition of TNF signaling

**DOI:** 10.1007/s00395-016-0573-2

**Published:** 2016-07-19

**Authors:** Alessandra Martire, Fikru B. Bedada, Shizuka Uchida, Jochen Pöling, Marcus Krüger, Henning Warnecke, Manfred Richter, Thomas Kubin, Susanne Herold, Thomas Braun

**Affiliations:** 1 Department of Cardiac Development and Remodelling, Max-Planck-Institute for Heart and Lung Research, Ludwigstrasse 43, 61231 Bad Nauheim, Germany; 2 Institute for Heart and Circulation Research, University of Witten/Herdecke, Alfred-Herrhausen-Straße 50, 58448 Witten, Germany; 3 Department of Cardiac Surgery, Schüchtermann-Klinik, Ulmenallee 11, 49214 Bad Rothenfelde, Germany; 4 Department of Cardiac Surgery, Kerckhoff-Clinic, Benekestrasse 2-8, 61231 Bad Nauheim, Germany; 5 Department of Internal Medicine II, Universities of Giessen and Marburg Lung Center, Klinikstr. 33, 35392 Giessen, Germany

**Keywords:** Mesenchymal stem cells, Proteomics, TNF-α, Fibrosis, Inflammation, Heart failure, Acute lung injury

## Abstract

Mesenchymal stem cells (MSC) have been used to treat different clinical conditions although the mechanisms by which pathogenetic processes are affected are still poorly understood. We have previously analyzed the homing of bone marrow-derived MSC to diseased tissues characterized by a high degree of mononuclear cell infiltration and postulated that MSC might modulate inflammatory responses. Here, we demonstrate that MSC mitigate adverse tissue remodeling, improve organ function, and extend lifespan in a mouse model of inflammatory dilative cardiomyopathy (DCM). Furthermore, MSC attenuate Lipopolysaccharide-induced acute lung injury indicating a general role in the suppression of inflammatory processes. We found that MSC released sTNF-RI, which suppressed activation of the NFκBp65 pathway in cardiomyocytes during DCM in vivo. Substitution of MSC by recombinant soluble TNF-R partially recapitulated the beneficial effects of MSC while knockdown of TNF-R prevented MSC-mediated suppression of the NFκBp65 pathway and improvement of tissue pathology. We conclude that sTNF-RI is a major part of the paracrine machinery by which MSC effect local inflammatory reactions.

**Electronic supplementary material:**

The online version of this article (doi:10.1007/s00395-016-0573-2) contains supplementary material, which is available to authorized users.

## Introduction

Mesenchymal stem cells (MSC) are multipotent cells that can be found in the stroma of various organs. MSC have been originally described as adherent, non-hematopoietic cells, which are present in the bone marrow of different species (reviewed by [25]). Bone marrow-derived MSC own a certain plasticity, which enables them to differentiate into osteogenic, chondrogenic, and adipogenic lineages [36]. Subsets of MSC have also been claimed to contribute to various other cell types from all the three germ layers including cardiomyocytes [14] but hard experimental evidence for this hypothesis is limited [3, 25, 37].

Shortly after description of reliable methods to isolate MSC clinical trials were launched to explore safety and efficacy of MSC treatments. In hallmark studies it was demonstrated that allogenic MSC have a potential to treat children with osteogenesis imperfecta although further studies indicated only a temporary short-term benefit (reviewed in [24]). Nevertheless, the concept that MSC might serve as a powerful “natural system for tissue repair” triggered the use of MSC as therapeutic agents in a variety of experimental models of tissue injuries. Examples include the treatment of chronic skin wounds, ischemic hearts, damaged brains, lungs, kidneys, and other organs (reviewed in [32]). In virtually all cases, the mechanism by which MSC exerted positive effects remained enigmatic. The finding, however, that the level of tissue engraftment did not correlate with the therapeutic benefit suggested that differentiation of MSC into functional parenchymal cells is irrelevant in most settings [11, 15, 26, 28, 33]. Hence, recent attention focused on the potential release of bioactive factors by MSC, which might alter the tissue microenvironment to stimulate organ-intrinsic progenitor cells or modulate other pathogenetically relevant processes such as inflammation (reviewed in [15, 25]). In fact, the immunomodulatory effect of MSC has been already described in initial transplantation experiments and thereafter found to be an active process based on the interaction with T cells and other cells of the immune system such as dendritic cells and macrophages (reviewed in [5, 15, 25, 32]). Several factors have been identified, which mediate these interactions in vitro but a thorough analysis of the molecules that modulate immune responses and inflammatory reactions in vivo proved to be difficult.

The tight regulation of pro- and anti-inflammatory processes is particularly important during development of heart failure and acute lung injury. It is assumed that the balance between pro- and anti-inflammatory mediators regulates the degree of the inflammatory response and thereby has a major impact on the course of the underlying disease [8, 13, 38]. We previously described that the cytokine receptor CCR2 is necessary for organ-specific homing of adult bone marrow-derived MSC to the heart in a transgenic mouse model of inflammatory dilative cardiomyopathy and into hearts damaged by ischemia/reperfusion. Both models are characterized by a high degree of mononuclear cell infiltration and inflammatory reactions, which might explain why MSC were efficiently attracted by a cytokine that is thought to be primarily involved in the attraction of macrophages [4]. The homing of MSC into diseased organs characterized by strong inflammatory reactions also raised the question whether MSC modulate the disease process probably by exerting immunosuppressive effects. We therefore started to explore the potential beneficial effects of MSC in models of inflammatory DCM and acute lung injury.

Here, we demonstrate that administration of MSC significantly improved cardiac function and survival along with an improvement of several morphological parameters. Furthermore, MSC administration attenuated LPS-induced lung injury suggesting that MSC provide beneficial effects in different organs suffering from inflammatory processes. To understand the molecular processes influenced by MSC, we performed a comprehensive analysis of the secretome of MSC and found that MSC release significant amounts of sTNF-RI, which were sufficient to inhibit the NFκB signaling pathway in vivo. Substitution of MSC by recombinant soluble TNF-R partially recapitulated the positive effects of MSC in the heart and lung indicating that release of sTNF-RI accounts for parts of the actions of MSC in an inflammatory environment.

## Materials and methods

### Antibodies and reagents

Antibodies directed against soluble tumor necrosis factor receptor I (sTNF-RI), lymphotoxin-α (LTα or TNFSF1) were purchased from R&D systems. Antibodies recognizing phospho p65 (Ser536; P-p65), phospho IκBα (Ser32; P-IκBα), and TNF receptor-associated factor 2 (Traf2) were obtained from Cell Signaling Technology. Anti-fibronectin, RalA (Ras-related protein) and pan-actin were from Becton–Dickinson. Antibodies directed against actinin and collagen VI were purchased from Sigma and Biomol, respectively. Enbrel (Etanercept) and pentoxifylline (PTX) were obtained from Amgen and Sanofi-Aventis, respectively. The MIP-2α ELISA kit was purchased from R&D systems.

### shRNA-mediated knockdown of TNF-RI

The knockdown of TNF-RI was accomplished using the MISSION shRNA system from Sigma–Aldrich. shRNA lentiviral plasmids (in pLKO.1-puro) with the clone IDs NM_011609.2-1538s1c1, NM_011609.2-996s1c1, and NM_011609.2-1652s1c1 were employed to generate lentiviral particles, which were used to infect MSC (MSC + TRkd) as described previously [4]. The efficacy of knockdowns was monitored by Western blot analysis before intravenous injection into recipient mice. The non-target shRNA control SHC002, which does not target a mouse or human mRNA was used as a negative control.

### Stem cell transplantation and drug treatment of wild type and transgenic mice

The generation of MCP-1 transgenic mice on FVB/N background, hereafter referred to as “TG”, has been described previously [16]. Wild type (WT) FVB/N mice were used as control in morphological and Western blot analyses. Housing and handling of mice were in accordance with the American Physiological Society guidelines for animal welfare and the Bioethical Committee of the District of Darmstadt, Germany. Long-term renewing adult bone marrow-derived multipotent mesenchymal stem cells (MSC) were isolated and transplanted into tail veins of recipient hosts as described previously [4]. Treatment with Enbrel was accomplished by subcutaneous injection at 10 μg/g body weight in saline solution while PTX was injected intraperitoneally at 8 μg/g body weight in saline solution. WT and TG mice were analyzed at 4 months of age by MRI followed by the first injection of MSC, Enbrel or PTX 2 weeks later. Injection of saline solution was used as a sham control. Three additional injections of MSC, Enbrel or PTX were done at weekly intervals as outlined in (Fig. S1A). At 6 month of age, mice were analyzed by MRI, western blot and immunofluorescence.

To induce acute lung injury, wild type mice C57/Bl6 mice were subjected to intratracheal instillation of 50 or 100 µg Lipopolysaccharide (LPS). Bronchoalveolar lavage fluid (BALF) was collected at indicated time points. Albumin leakage was determined as described previously [12]. MSC, Enbrel and PTX were applied either intratracheally (i.t.) or intraperitoneally (i.p.). Polymorphonuclear leukocyte numbers were determined after Pappenheim staining of cytospin preparations as described previously [12].

### Magnetic resonance imaging (MRI) studies

Cardiac MRI measurements were performed on a 7.0 T Bruker Pharmascan, equipped with a 300 mT/m gradient system, using a custom-built circularly polarized birdcage resonator and the Early Access Package for self-gated cardiac Imaging (Intragate, Bruker) [18]. MRI data were analyzed using Qmass digital imaging software (Medis, Leiden, Netherlands) as described previously [20].

### Histology and immunofluorescence

Cryosections for histological and immunohistochemical analyses were prepared from snap frozen hearts and subjected to Masson’s trichrome according to the manufacturer’s instructions (Sigma–Aldrich). Immunofluorescence was accomplished using 7 μm thick cryosections after 4 % paraformaldehyde fixation for 10 min. After overnight incubation with primary antibodies, slides were washed with PBS and incubated with anti-rabbit Alexa 488- or Alexa 594 -conjugated secondary antibodies (Alexa), washed with PBS and counterstained with FITC- or TRITC-conjugated phalloidin (Sigma) to visualize actin [20]. Nuclei were visualized by incubation with DRAQ5™ (Alexis biochemicals) for 15 min. Omission of the first antibody served as negative control.

### Mass spectrometry analysis

MSC were grown in SILAC medium for at least five cell divisions as described previously to ensure complete labeling of cells [17]. Cells were washed six times in serum-free medium, and incubated in serum-free medium. The conditioned medium containing the secreted proteins was collected, filtered using a 0.45 μm filter (Millipore, Bedford, MA) and concentrated using a 3000 Da molecular mass cutoff spin column, Centriprep (Millipore). Processing of protein samples and mass spectrometry was performed as described [7] using an LTQ-Orbitrap (Thermo Fisher Scientific) combined with an Agilent 1200 nanoflow HPLC system. The mass spectrometer was operated in the data-dependent mode to automatically measure full MS scans and MS/MS spectra. Peptides were identified by searching in the International Protein Index sequence database (mouse IPI, version 3.24) using MaxQuant (version 1.5.1.0), which performs peak lists, SILAC quantification and false positive rate determination. Go-terms were implemented using Perseus (version 1.5.0.15).

### Preparation of protein extracts and Western blot analysis

Protein extracts were prepared using standard procedures [20]. Ten micrograms of protein extracts was separated on 4–12 % SDS–PAGE gradient gels and processed as previously described [40]. Immunoreactive proteins were quantified with the Quantity One software (BioRad) after visualization on the VersaDoc system (BioRad). Relative concentrations of secreted cytokines were analyzed using the RayBio cytokine antibody array 3.1 as described by the supplier (RayBiotech, Norcross, USA).

### Statistics

The following tests were used to investigate statistical significance: (1) for three groups one-factor ANOVA and subsequent Bonferroni multiple comparisons or Kruskal–Wallis test and subsequent Dunn’s multiple comparisons in the case of missing normality or heterogeneous variance of the data; (2) for two groups unpaired *t* test or Mann–Whitney *U* test in the case of missing normality or heterogeneous variance of the data. For the survival analysis the Kaplan–Meier method was used. Differences in survival between groups were tested using the log-rank test and Pearson’s *χ*^2^ test, respectively. *p* values less than 0.05 were considered statistically significant. No statistical method was used to predetermine sample size.

## Results

### MSC improve cardiac functions and extend the lifespan of MyHC-MCP-1 transgenic mice suffering from inflammatory dilative cardiomyopathy

**Fig. 1:**
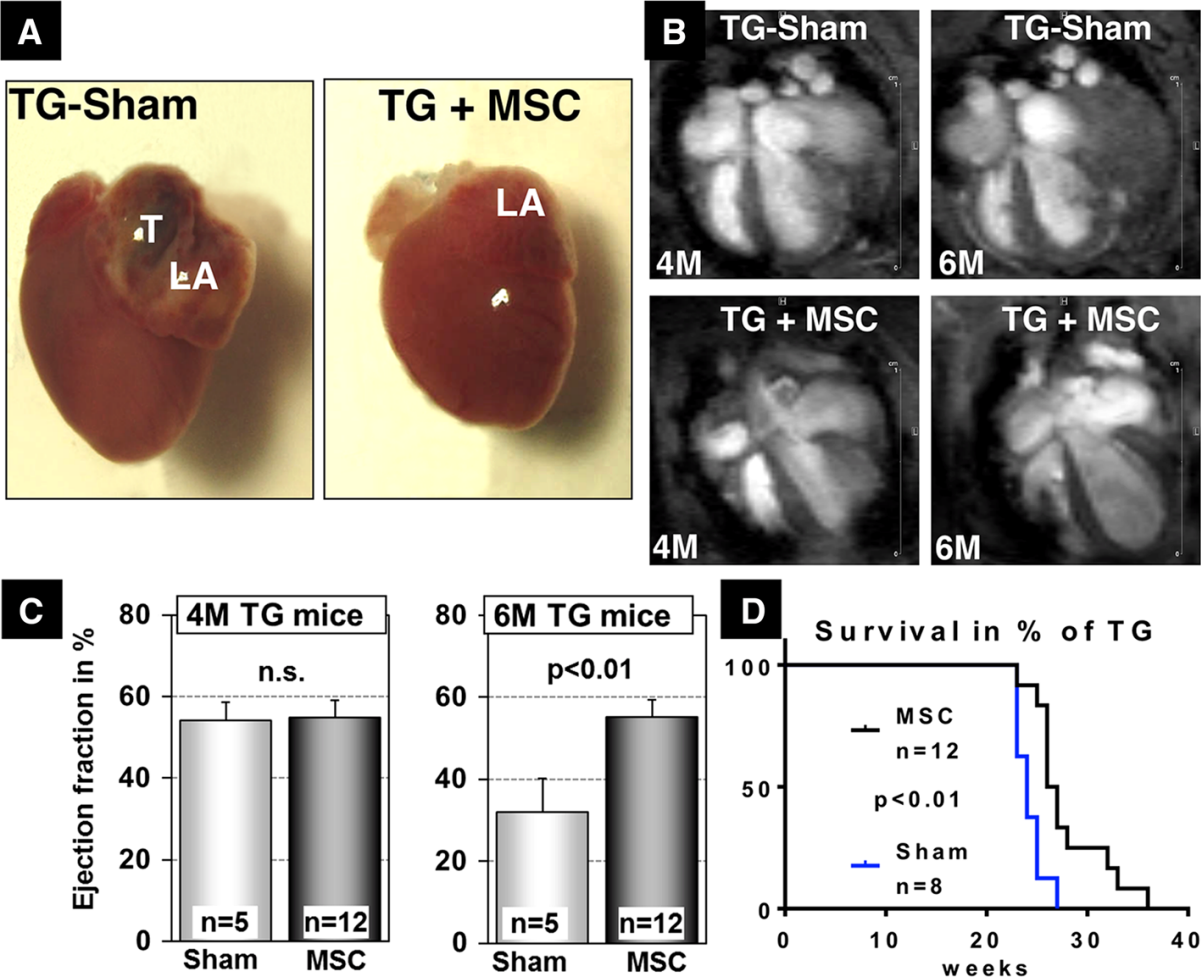
Improvement of cardiac function and lifespan extension after injection of MSC into mice with inflammatory DCM (TG). **a** Macroscopic view of hearts dissected from 6-month-old αMyHC-MCP-1 mice, which received sham-treatment (TG-Sham) or MSC injections (TG + MSC). The lack of thrombus (T) formation in the *left atrium* (LA) is clearly visible. **b** Injection of MSC prevented the decline in cardiac performance and ventricular dilatation of hearts of αMyHC-MCP-1 mice. MRI images of TG animals at 4 and 6 months of age with and without treatment are shown. **c** Statistical evaluation of improved ejection fraction at 4 and 6 months. **d** Survival curve of αMyHC-MCP-1 mice (TG) demonstrating significant extension of the lifespan after treatment with MSC

**Fig. 2:**
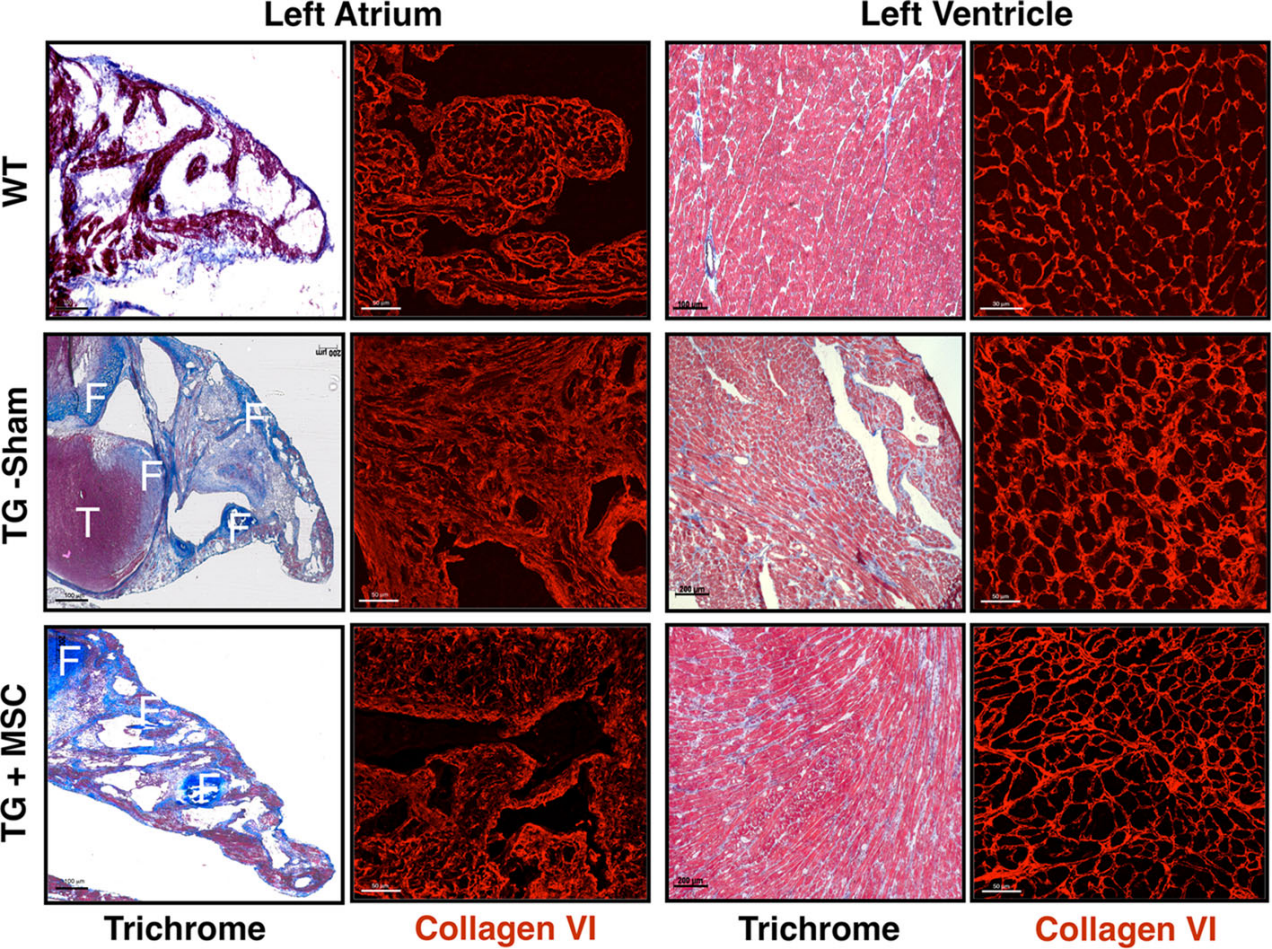
Reduced cardiac fibrosis and thrombus formation after intravenous injection of MSC into αMyHC-MCP-1 (TG) mice. Masson’s trichromic and collagen VI staining of sections from *left atria* and ventricles of TG mice, which received sham-treatment or MSC injections. Sections from WT mice are shown for comparison. Masson’s trichrome staining reveals reduced fibrosis in stem cell-treated TG mice (TG + MSC) in comparison to sham TG (TG-Sham) and wild-type mice (WT). Collagen fibers (F) are stained blue and muscle red. A large thrombus (T) is visible in sham-treated TG, which is absent in stem cell-treated mice. Immunofluorescence images of collagen VI demonstrate improved architecture of the extracellular matrix and a reduced collagen deposition in stem cell-treated TG mice. *Scale bars* 100 μm

Intrigued by our previous observation that MSC efficiently home into diseased hearts characterized by strong inflammatory reactions [4] we asked whether local accumulation of MSC modulates inflammatory signaling processes in the myocardium. We decided to focus our investigations on the αMyHC-MCP-1 model. αMyHC-MCP-1 mice develop inflammatory cardiomyopathy in a time dependent manner [16] and attract MSC into the heart after intravenous administration [4]. Cardiac functions of αMyHC-MCP-1 mice are still within the physiological range at 4 months but deteriorate rapidly thereafter, leading to congestive heart failure followed by premature death usually at 6 months [16, 34]. To test the ability of MSC to improve the condition of αMyHC-MCP-1 mice, we performed four weekly injections of MSC starting 2 weeks after an initial assessment of the cardiac function by magnetic resonance imaging (MRI) at 4 months of age (Fig. S1A). Two month after initiation of the regimen cardiac functions the same animals were analyzed again by MRI followed by morphological and biochemical analyses. Sham-treated αMyHC-MCP-1 mice (*n* = 5) showed left atrial (LA) cavity dilatation (Fig. 1a, b), thrombus formation in the LA (Figs. 1a, 2) and a decrease of the ejection fraction (EF) from 54 % at 4 month to 32 % at 6 month (Fig. 1c). In contrast, animals treated four times with 5.0 × 10^5^ MSC (*n* = 12) did not show a decline of ejection fractions as determined by MRI (Fig. 1c). Moreover, dilation of the LA and thrombus formation were significantly reduced in treated compared to control animals (Figs. 1a, b, 2). Importantly, we also observed a significant extension of the lifespan of αMyHC-MCP-1 mice. All sham-treated animals died before the age of 27 weeks whereas some MSC-treated αMyHC-MCP-1 mice survived until the age of 36 weeks. The average lifespan increased from 24 ± 0.5 (untreated) to 28 ± 1.5 weeks (treated) (*n* = 8 for sham-treated and *n* = 12 for MSC-treated mice, *p* < 0.01) (Fig. 1d).

### Administration of MSC improves cardiac morphology in a model of inflammatory dilative cardiomyopathy

**Fig. 3:**
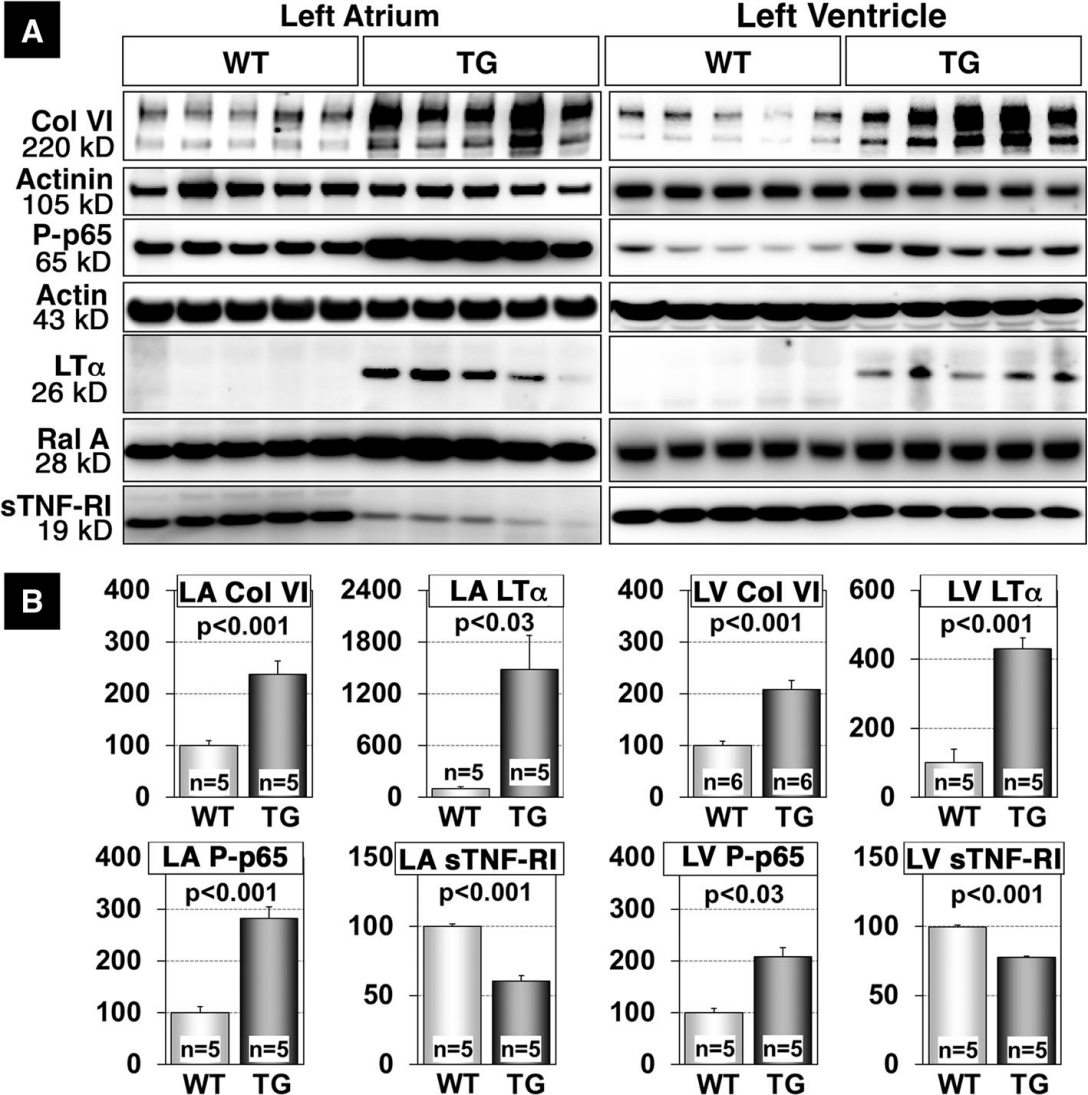
Activation of the TNF-signaling cascade in mice with inflammatory dilated cardiomyopathy. **a** Western blot analyses of tissue lysates from *left atrium* and *left ventricle* of 6-month-old wild-type (WT) and αMyHC-MCP-1 mice (TG) are shown. TG mice express increased amounts of Collagen VI (Col VI), phosphorylated-p65 (Ser536; P-p65) and lymphotoxin-α (LTα) in the atrium and in the ventricle. The expression level of sTNF-RI is decreased in the atrium as well in the ventricle of TG mice. RalA (Ras-related protein) served as loading control. **b** Statistical analysis reveals stronger effects in the atrium compared to the ventricle of TG mice

Next, we investigated the morphology of the myocardium of αMyHC-MCP-1 mice at 6 month of age in MSC-treated and control animals. Masson´s trichrome staining revealed a diminished fibrosis in the LA and in the left ventricle (LV) of MSC-treated mice (Fig. 2). Confocal imaging of sections stained with an antibody directed against collagen VI indicated reduced collagen deposition and improved tissue architecture in treated αMyHC-MCP-1 mice, which went along with reduced expression of ANP (Figs. 2, 3, S1B). Both the LA and LV of treated αMyHC-MCP-1 mice were characterized by moderately reduced infiltration with CD45-positive inflammatory cells (Fig. S2).

Re-establishment and maintenance of blood supply are of pivotal importance for damaged tissues. We therefore investigated the distribution of capillary vessels in MSC-treated and control mice. Staining with BS-1, a marker for endothelial cells, revealed an increase of the capillary density in the LA but not the LV of treated mice (*n* = 5 for control, *n* = 12 for treated mice) (Figs. S3, S4). The uneven increase of capillary density suggests that this effect is most likely caused by indirect mechanisms and not by differentiation of MSC to endothelial cells or direct stimulation of endothelial cell growth since we did not find a preferential accumulation of transplanted MSC within the left atrium (data not shown).

### MSC release a large number of biologically active molecules

Our previous studies revealed that genetically labeled MSC injected into αMyHC-MCP-1 mice either formed small clusters or were scattered throughout the myocardium [4]. Careful morphological analysis indicated that virtually none of these cells expressed cardiomyocyte markers such as sarcomeric actinin and troponin, although a minor fraction was positive for the endothelial cell marker BS-1 (Fig. S4 and data not shown). The relatively low number of BS-1 positive cells suggested that most, if not all, of the positive effects of MSC were mediated by the local release of bioactive molecules. We therefore decided to investigate the secretome of MSC using SILAC-based mass spectrometry. Culture supernatants were sampled at 4, 12, and 18 h resulting in identification of a total of 3066 proteins with at least 2 peptides and 1 unique peptide (Table S1). Next, we performed a Gene Ontology (GO)-annotation analysis to identify proteins that were actively released from MSC and not passively due to death of cells during the sampling period. Based on the annotation we identified 585 proteins, which are localized at the plasma and basement membrane, present in the cytosol, or secreted, including several C–C– and C–X–C–motif chemokines, SDF1 and SDF2, metalloproteinase inhibitors, and TNF-R superfamily member 11B (Fig. S5; Table S1). Of these, 101 proteins were differentially enriched (>1.5-fold change) in the secretome of MSC vs the mesenchymal cell line 10T1/2 while the remaining 484 proteins were expressed at similar or even higher levels by 10T1/2 cells. Notably, six chemokine related proteins showed increased secretion from MSC compared to 10T1/2 cells (Fig. S5; Table. S1). Despite the excellent sensitivity of modern mass spectrometry, it is still not possible to detect all low abundant proteins. We therefore complemented our mass spectrometry-based secretome measurements using protein cytokine arrays. Analysis of conditioned cell culture supernatants from MSC with the mouse cytokine array 3.1 (RayBiotech) that detects 62 cytokines validated the release of 6 proteins (Axl, Cx3cl1, Igfbp5, Timp1, Vcam1, Cxcl12) identified in the mass spectrometry experiment (Table S1) and additionally recognized TARC/CCL17 (thymus- and activation-regulated chemokine) and sTNF-RI (soluble TNF receptor I) (Fig. S5).

### Heart failure in αMyHC-MCP-1 is associated with activation of the TNF/NFκB signaling pathway and downregulation of sTNF-RI

The improvement of cardiac functions of αMyHC-MCP-1 suffering from inflammatory DCM by MSC suggested that MSC pathogenetically influence relevant processes in the diseased heart probably by a mechanism that controls inflammatory responses. Such postulate would also fit to the proposed immunomodulatory function of MSC [5, 15]. One of the potentially most interesting molecules in this respect is sTNF-RI, which was identified during our secretome analysis of MSC (Fig. S5 and data not shown). sTNF-RI is the soluble form of the receptor of tumor necrosis factors, which is present at the surface of many cells. sTNF-RI binds TNF-α and lymphotoxin-α (LTα or TNFSF1) thereby inhibiting their activities, although it has been shown that sTNF-RI also stabilizes TNF and increases its half life under certain conditions [1, 2].

To investigate whether TNF-related processes are active in αMyHC-MCP-1 mice, we analyzed several components of the TNF-pathway by Western blot analysis. We detected a strong expression of LTα in the LA and LV of αMyHC-MCP-1 mice, which was completely absent in wild-type control mice in the LA and LV (Fig. 3a, b). Interestingly, we also found a significant downregulation of sTNF-RI, which was strongly expressed in normal wild-type hearts but was only faintly present in transgenic animals in the LA and LV (Fig. 3a, b). Moreover, phosphorylation of NFκBp65, one of the main intracellular signal transducers of TNF signaling, was strongly upregulated in the LA and LV of transgenic mice indicating activation of the inflammatory TNF-pathway (Fig. 3a, b). This observation is consistent with the strong induction of the NFκBp65 targets Traf2 (TNF receptor-associated factor-2) and fibronectin (Fig. S6A, B). Furthermore, we detected increased phosphorylation of the NFκBp65 inhibitor IκBα at S32, which leads to its proteasome-mediated degradation causings derepression of NFκBp65 signaling (Fig. S8A, B). Enhanced activation of inflammatory responses in transgenic animals went along with deposition of collagen VI in the LA and LV corroborating our histological findings (Fig. 2). Statistical analysis indicated that changes in the activation of the TNF-pathway were more pronounced in the atrium than in the ventricle (Fig. 3a, b).

### Transplantation of MSC restores sTNF-RI concentrations and inhibits NFκB activation in the myocardium of αMyHC-MCP1 mice

**Fig. 4:**
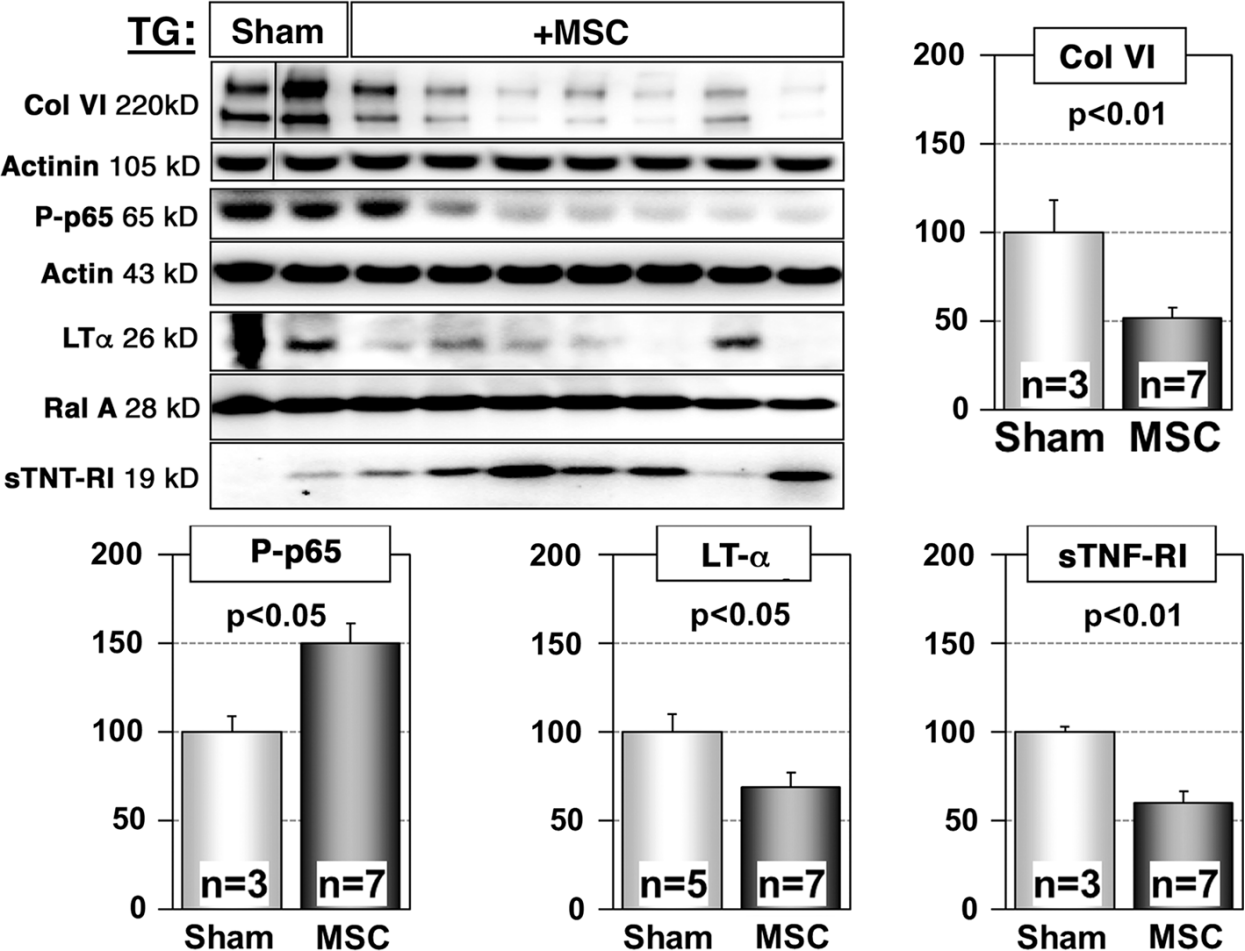
Inhibition of TNF signaling in hearts of αMyHC-MCP-1 (TG) mice after administration of MSC. Western blot analyses of atria from 6-month-old transgenic animals (TG), which received sham-treatment or MSC injections. Injection of MSC resulted in reduced presence of collagen VI (Col VI), phosphorylated-p65 (Ser536; P-p65) and lymphotoxin-α (LTα) but increased levels of sTNF-RI. RalA (Ras-related protein) served as a loading control. The statistical analysis of different biological replicates is shown

We next examined whether transplantation of MSC affects the TNF/NFκB system. Western blot analysis of the myocardium of αMyHC-MCP1 mice, which received MSC injections, showed a strong inhibition of phosphorylation (activation) of NFκBp65 at Ser536 compared to sham-treated controls (Fig. 4) (*n* = 3 for sham controls, *n* = 7 for treated mice, *p* < 0.05). Similarly, treatment with MSCs reduced levels of phosphorylated IκBα in αMHC-MCP1 hearts, which prevents its degradation thereby inhibiting NFκBp65 signaling. In addition, we observed decreased expression of the NFκBp65 targets fibronectin (Fn1) and Traf2 by immunofluorescence staining and Western blot analysis (Fig. S8) which further corroborated the inhibition of NFκBp65 signaling. Transplantation of MSC also increased levels of sTNF-RI in the hearts of αMyHC-MCP1 mice, which nearly reached levels seen in wild-type controls (Fig. 4). Interestingly, the expression of LTα dropped after transplantation of MSC (Fig. 4) suggesting that MSC inhibited TNF-feedback loops, which are believed to contribute to increased amounts of LTα in inflamed organs [35]. The inhibition of NFκBp65 activation in MSC transplanted αMyHC-MCP1 mice was associated with decreased deposition of collagen VI and fibronectin (Fig. 4, S6). In contrast, MSC infected with a lentivirus expressing a siRNA directed against TNF-RI failed to block NFκBp65 activation, collagen VI deposition, LTα expression, and failed to restore sTNF-RI expression (Fig. S7).

### Recombinant soluble TNF receptor partially mimics effects of MSC

**Fig. 5:**
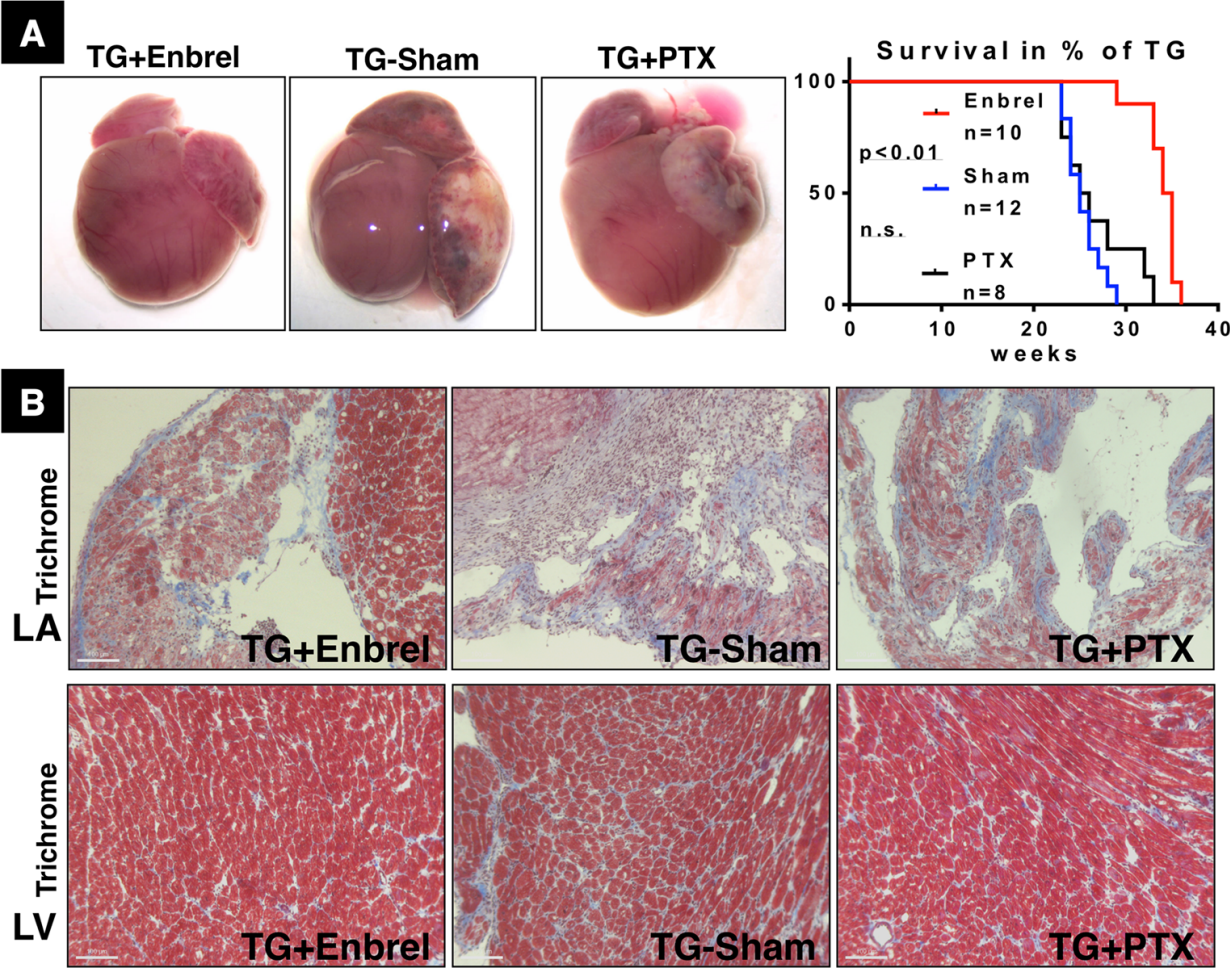
Administration of recombinant soluble TNF receptor (Enbrel) mimics effects of MSC in αMyHC-MCP-1 mice. **a** Macroscopic views of hearts dissected from αMyHC-MCP-1 mice (TG) after Enbrel, Pentoxifylline (PTX), or sham-treatment at 6 months of age. Treatment with Enbrel reduced atrial and ventricular dilatation and prevented thrombus formation. Statistical analysis of the survival time of TG mice after treatment with Enbrel and Pentoxifylline is shown on the *right*. **b** Masson’s trichrome staining shows reduced fibrosis in the *left atrium* and *left ventricle* of Enbrel- or PTX-treated animals. Collagen fibers are stained *blue* and muscle is *red*. *Scale bars* 100 μm

The relation between sTNF-RI release by MSC, inhibition of NFκBp65 activation, and inhibition of LTα expression in hearts of αMyHC-MCP-1 mice strongly suggested that disruption of TNF signaling by sTNF-RI is at least partially responsible for the positive effects of MSC on cardiac function and survival. To prove this hypothesis we tried to mimic the effects of MSC by administration of Enbrel (also known as Etanercept), a recombinant protein that consists of two extracellular domains of the TNF-RII receptor (p75) fused to IgG1, and pentoxifylline (PTX), which increases the intracellular concentration of cAMP cyclic adenosine monophosphate resulting in decreased TNF-α production. Treatment of αMyHC-MCP-1 mice with Enbrel led to significant extension of the average lifespan from 25 weeks in the sham-treated group to 34 weeks in the Enbrel group (*n* = 12 control mice, *n* = 10 Enbrel-treated mice, *p* < 0.001) (Fig. 5a). In contrast, no extension of the lifespan was monitored in the PTX group (*n* = 8 pentoxifylline-treated mice) (Fig. 5a). αMyHC-MCP-1 mice treated with either Enbrel or PTX showed a reduced degree of thrombus formation and LA dilatation at 6 months of age, although this effect was more pronounced in the Enbrel compared to the PTX group (Fig. 5a). Masson’s trichrome staining revealed a reduced fibrosis in the LA and LV (Fig. 5b) and a reduced collagen deposition after both therapies (*n* = 13 for each group). Similar to the LA and LV of MSC-treated mice (Fig. S2), we also observed a reduced accumulation of CD45-positive inflammatory cells in the LA and LV of Enbrel- and PTX-treated animals (Fig. S8). Moreover, we found an increase of the capillary density in the LA but not in the LV of Enbrel-treated mice (Fig. S3). No increase in the capillary density was detected in the PTX group (*n* = 13 for each group) (Fig. S3). Surprisingly, assessment of cardiac function by MRI did not reveal a consistent increase of the ejection fraction in Enbrel- or PTX-treated animals compared to the control group (data not shown).

**Fig. 6:**
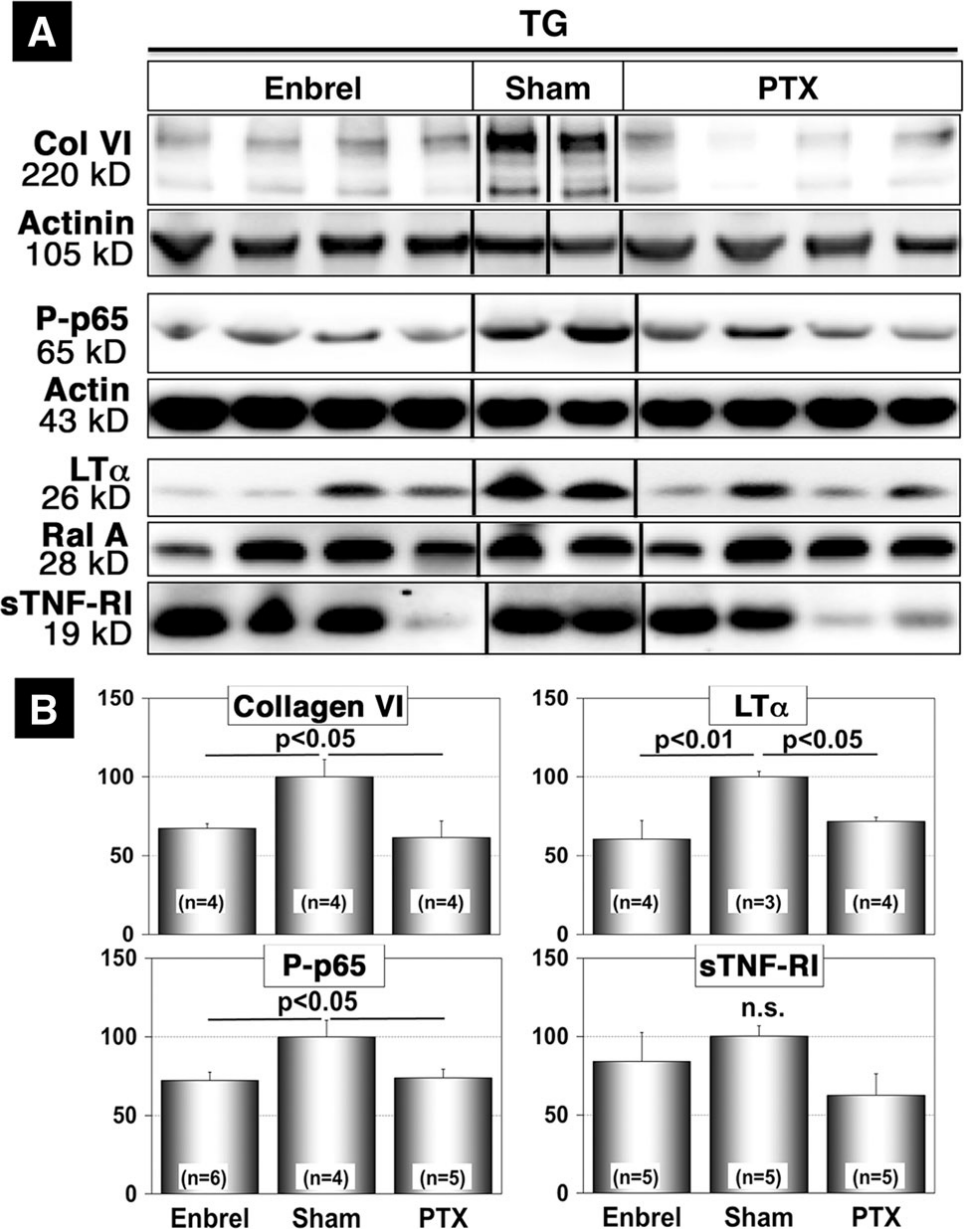
Administration of recombinant soluble TNF receptor (Enbrel) mimics several biochemical effects of MSC in αMyHC-MCP-1 mice. **a** Western blot analyses of tissue lysates from the *left atrium* of 6-month-old αMyHC-MCP-1 mice (TG) after Enbrel (TG + Enbrel), Pentoxifylline (TG + PTX), or sham-treatment (TG-Sham) are shown. Treatment with Enbrel or PTX decreased expression of collagen VI (Col VI) and LTα and resulted in reduced phosphorylation of NFκBp65 (Ser536; P-p65). Please note that treatment with Enbrel (unlike treatment with MSC) does not result in increased expression of sTNF-RI while treatment with PTX resulted in a further (non-significant) reduction of sTNF-RI levels. RalA (Ras-related protein) served as a loading control. **b** Statistical analysis of Western blots shown in (**a**)

To investigate whether improvement of cardiac morphology and lifespan extension after Enbrel treatment was associated with a reduced activation of the NFκB pathway, we performed a Western blot analysis. We detected a significant reduction of NFκBp65 phosphorylation in Enbrel- and PTX-treated animals (*n* = 6, 5, 4 for Enbrel-treated, PTX-treated, and sham-treated animals, respectively, *p* < 0.05), which was associated with reduced collagen VI deposition and reduced concentrations of LTα (Fig. 6a, b). Although treatment with Enbrel and PTX mimicked several effects of transplanted MSC, we did not observe an increase of sTNF-RI concentrations, which might indicate that sTNF-RI was directly derived from MSC. Taken together, our experiments revealed that injection of recombinant soluble TNF receptor recapitulated many, but not all effects of MSC suggesting that inhibition of the TNF/NFκB pathway is responsible for a major part of the beneficial effects of MSC in inflammatory DCM.

### Administration of MSC reduces LPS-induced inflammatory lung injury

**Fig. 7:**
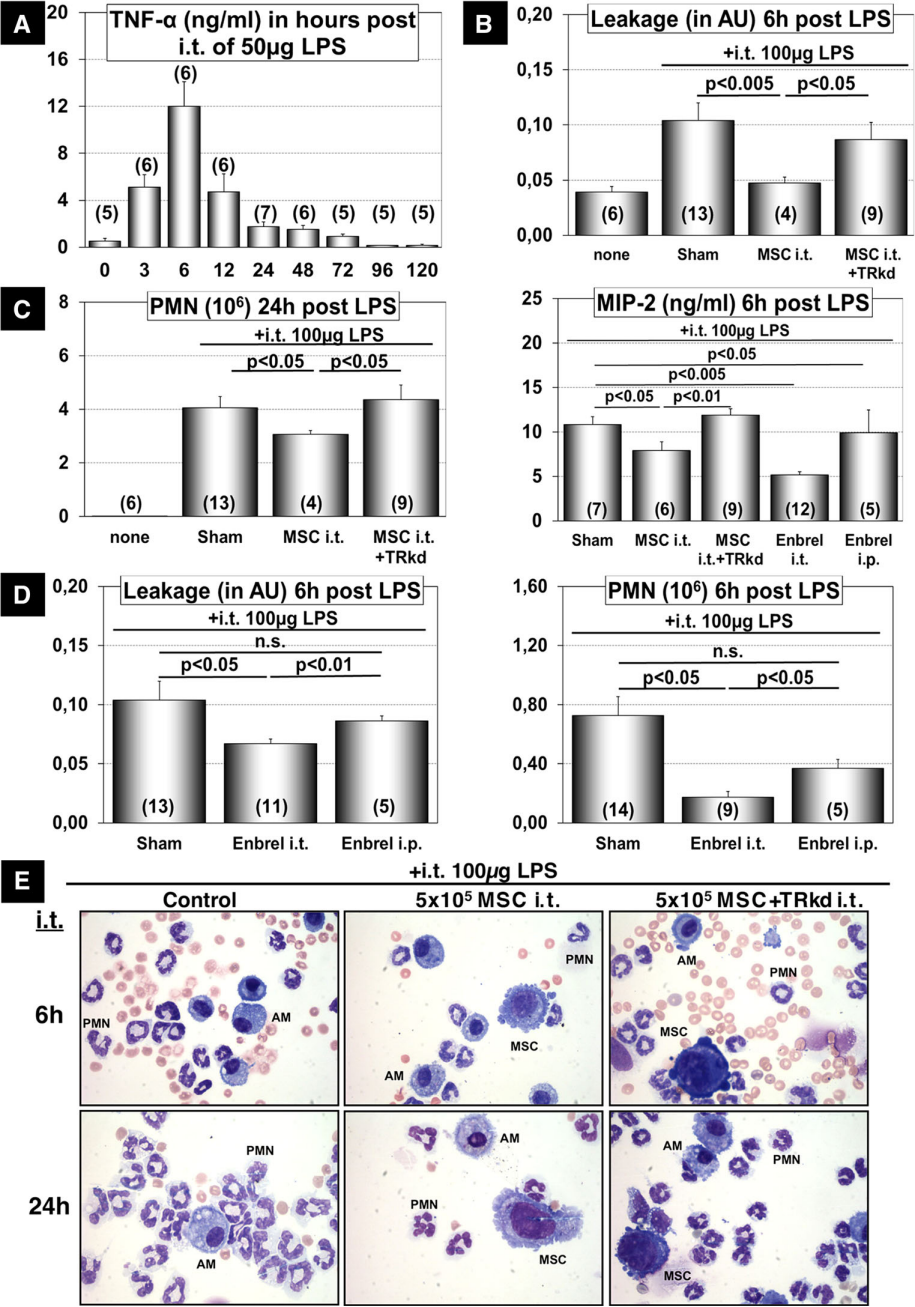
Attenuation of LPS-induced ALI after administration of MSC or Enbrel. **a** Time course of TNF-α concentrations in the bronchoalveolar lavage fluid (BALF) of mice after intratracheal instillation of LPS. **b** Intratracheal administration of MSC reduced alveolar leakage in LPS-induced ALI. MSC in which sTNF-RI was knocked down (MSC + TRkd) did not show significant effects compared to sham-treated animals. **c** Administration of WT MSC or Enbrel but not MSC + TRkd reduced the concentration of MIP-2α and the number of polymorphonuclear cells (PMN, mainly neutrophils) in the BALF. **d** Intratracheal instillation (i.t.) but not intraperitoneal injection (i.p.) of Enbrel reduced albumin leakage and accumulation of PMN in the BALF during LPS-induced ALI. **e** Pappenheim staining of cytospin preparations from the BALF of mice with LPS-induced ALI after administration of wild-type MSC or MSC + TRkd. Treatment with WT MSC but not MSC + TRkd reduced alveolar PMN accumulation and improved the barrier function as indicated by reduced alveolar hemorrhage

TNF signaling also plays a major role in the pathophysiology of lung diseases such as acute lung injury (ALI) and chronic obstructive pulmonary disease (reviewed by [27]). To determine whether the release of sTNF-RI by MSC represents a general principle that might explain the beneficial role of MSC in the suppression of inflammatory processes, we induced ALI through intratracheal administration of the bacterial endotoxin Lipopolysaccharide (LPS). In agreement with previous findings we detected a more than 20-fold increase of TNF-α concentrations by ELISA in the bronchoalveolar lavage fluid (BALF) within 6 h after intratracheal instillation of 50 µg LPS compared to sham-treated controls (Fig. 7a). Furthermore, we observed a robust recruitment of polymorphonuclear leukocytes (PMN) (Fig. 7c) into the alveolar space at 6 and 24 h after LPS administration, which was accompanied by leakage of FITC-labeled albumin from the vascular into the alveolar compartment, indicative of barrier function loss [12] (Fig. 7b). The damage of the alveolar epithelial barrier was also reflected by the presence of numerous erythrocytes in the BALF (Fig. 7e). Importantly, intratracheal instillation of 5 × 10^5^ MSC improved LPS-induced alveolar epithelial barrier leakage and reduced accumulation of PMN and the concentration of macrophage inflammatory protein 2α (MIP-2α), a PMN recruiting cytokine, in the BALF (Fig. 7b, c). In contrast, administration of MSC, in which the TNF-RI was knocked down by shRNA (MSC + TRkd), did not correct any parameters of ALI. The number of PMN (3.1 × 10^6^ PMN in MSC-treated animals vs 4.4 × 10^6^ PMN in MSC + TRkd, *p* < 0.05) as well as alveolar leakage (0.05 AU in MSC-treated mice vs 0.09 AU in MSC + TRkd treated animals; *p* < 0.05) did not show any significant differences compared to sham-treated mice (Fig. 7b, c). Similarly, the concentration of MIP-2α did not decline in the BALF of mice that received MSC + TRkd (Fig. 7c). To further prove that sTNF-RI is a major mediator of the positive effects of MSC during ALI, we administered Enbrel instead of MSC releasing sTNF-RI. We found that Enbrel recapitulated most effects of MSC in mice with ALI including reduction of albumin leakage, reduction of PMN numbers, and decrease of MIP-2α in the BALF (Fig. 7c, d). We noted that it was more effective to administer Enbrel i.t. rather than i.p., which might be explained by a better bioavailability of the protein within the damaged tissue (Fig. 7d). Taken together, our data strongly suggest that MSC exert protective effects in different inflammatory conditions by release of sTNF-RI.

## Discussion

Transplantations of MSC are used in numerous preclinical and clinical studies to enhance repair of damaged tissue and to attenuate immunological dysfunctions. In a recent review 344 registered clinical trials were counted that are designed to evaluate MSC therapy for multiple diseases [41]. Yet, the mechanisms behind clinical benefits are still mostly enigmatic. Initially, the therapeutic concept for treatment was based on the assumption that MSC differentiate and replace damaged tissue (see for example [30] and [29]). This view has changed to implement findings that MSC or their progenies do neither engraft nor differentiate efficiently within host tissues but nevertheless induce clear effects (see [11] for an example). Nowadays, most researchers favor the view that MSC act via different paracrine mechanisms [21] or exert immunomodulatory activities [5, 15]. Similarly, we did not find convincing evidence for differentiation of MSC into parenchymal cells when we traced the fate of injected cells during the course of our study, emphasizing the concept of paracrine signaling as the main basis for effects of MSC.

We have previously described that subsets of MSC rely on the cytokine receptor CCR2 and its intracellular receptor FROUNT for accumulation in hearts damaged by inflammation or ischemia/reperfusion [3, 4]. Here, we demonstrate that administration of MSC improves cardiac morphology/function and extends the lifespan of mice suffering from inflammatory cardiomyopathy. Similarly, MSC alleviated several signs of ALI suggesting that multiple inflammatory conditions can be targeted by MSC treatment. Analysis of the secretome of MSC revealed that MSC release sTNF-RI, thereby, blocking a major inflammatory pathway. We reason that this mechanism is responsible for the beneficial effects of MSC in inflammatory cardiomyopathy and ALI based on three arguments: (1) administration of MSC restored sTNF-RI concentrations, reduced the amount of LTα, and inhibited activation of NFκBp65 in the heart, which all can be explained by inhibition of TNF-R signaling. (2) Replacement of MSC by recombinant sTNF-R recapitulated most of the effects of MSC in inflammatory cardiomyopathy and ALI. (3) Knockdown of sTNF-RI in MSC resulted in the failure of MSC to inhibit TNF-R signaling and abrogated beneficial effects. Despite this accumulated evidence, we do not claim that all effects of MSC can be explained solely by the release of sTNF-RI, in particular since MSC secrete a large variety of additional bioactive molecules such as cytokines, thrombospondins, matrix metalloproteinase inhibitors, IGF-binding proteins, and SDF-1, which might contribute to the observed effects of MSC. In this context, it is interesting to note that treatment with Enbrel improved cardiac morphology and extended the lifespan but did not improve the cardiac ejection fraction. This observation might suggest the involvement of additional factors released by MSC but also indicates that the increased ejection fraction after administration of MSC is not instrumental for the survival of the animals.

Since both inflammatory monocytes/macrophages and MSC express CCR2, it is tempting to speculate that the release of CCL2 in damaged tissue will summon both inflammatory cells as well as MSC, which might counteract excessive inflammatory responses. Interestingly, we found significant concentrations of sTNF-RI in healthy hearts, although we do not know the relative contribution from circulating or tissue-resident MSC and other cell types. Such local levels of sTNF-RI might act as a safeguard to restrict TNF signaling in healthy hearts. We detected a significant drop of sTNF-RI concentrations during the course of inflammation, which was normalized by administration of MSC thereby partially restoring a potential countermeasure. Since MSC release sTNF-RI, it seems reasonable to assume that increased levels of sTNF-RI directly originate from recruited MSC although we cannot exclude a more indirect effect. Increased concentrations of sTNF-RI might affect both TNF-α and LTα. So far, it was assumed that TNF-α is the dominant ligand for TNF receptors in inflammatory cardiomyopathy [23]. Hence, the marked increase of LTα in the LV and even more pronounced in the LA was surprising. LTα is a rare cytokine, which is expressed by activated T cells, B cells, natural killer cells, and some non-hematopoietic cells while TNF-α is derived from invading monocytes/macrophages and from many resident cardiac cell types. On the other hand, TNF-α and LTα bind to the same receptors [p55 (TNF-RI) and p75 (TNF-RII)], which are present on almost every cell type in the cardiovascular system, and elicit a similar spectrum of activities [6]. Since TNF-α causes an increase of LTα expression in inflamed organs probably by promoting T-cell infiltration [35], the suppression of LTα expression by administration of MSC might reflect a general inhibition of inflammatory responses. This conclusion would also fit well to the reduced presence of inflammatory cells and the improved cardiac morphology in MSC-treated hearts. In addition, a part of the beneficial effects of MSCs on the myocardium of αMyHC-MCP-1 mice, which is characterized by reduced capillary density, might be due to pro-angiogenic activities of MSC. In fact, MSCs have been claimed to support angiogenesis either by enhancing endothelial cell growth and survival [39] or by differentiation into endothelial cells [31], although we did not obtain clear evidence for a significant contribution of transplanted MSC to endothelial cells in our experiments.

A link between TNF-signaling and chronic heart failure has been proposed some time ago [19], although it took a while to translate these findings into a clinical application. Initially, treatment of chronic heart failure with Enbrel was described to exert beneficial effects [9, 10] but a large randomized trial (“RECOVER” and “RENAISSANCE”) was stopped because primary endpoint results yielded no positive effects [22]. In contrast to our well-defined preclinical animal model of inflammatory DCM, human patients participated in the clinical study, who developed chronic heart failure due to a wide spectrum of different etiologies. Other major differences were the inclusion of patients with different degrees of chronic heart failure (NYHA class II–IV) and the premedication of human patients with a number of different drugs [22]. Our animal data suggest that patients suffering from inflammatory DCM might benefit from the treatment with MSC or Enbrel and that it is instrumental to initiate the treatment at early disease stages before severe damages have accumulated. In our comparative study, the use of MSC was superior to the treatment with Enbrel and PTX in respect to tissue morphology and heart function. Two reasons might account for these differences: (1) MSC release numerous different factors that might act together with sTNF-RI and increase its effectiveness. (2) MSC accumulate within diseased regions of the heart which will increase local concentrations of sTNF-RI and reduce systemic side effects.

Taken together we have demonstrated that administration of MSC suppressed inflammatory processes in the heart and in the lung most likely by inhibition of NFκBp65 signaling via sTNF-RI. In both tissues, disease parameters were improved by treatment with MSC including extended survival of mice suffering from inflammatory DCM. It seems likely that improved homing of MSC coupled with enhanced release of sTNF-RI or similar molecules will further increase treatment efficiency. We reason that the usage of MSC with anti-inflammatory properties presents an attractive approach to treat human patients suffering from chronic inflammatory heart and lung diseases.

## Electronic supplementary material

Below is the link to the electronic supplementary material.

Supplementary material 1 (PDF 9388 kb)

Supplementary material 2 (XLSX 1804 kb)
