# Supplementary material for: Mesenchymal stem cells attenuate inflammatory processes in the heart and lung via inhibition of TNF signaling
Source: Basic Res Cardiol. 2016 Jul 19;111(5):54. doi: 10.1007/s00395-016-0573-2 (PMC4951509; doi:10.1007/s00395-016-0573-2)
Supplement: Supplementary file 1 — Supplementary material 1 (PDF 9388 kb) [file 395_2016_573_MOESM1_ESM.pdf]

Supplementary Material

Mesenchymal stem cells attenuate inflammatory processes in the heart and lung via inhibition of TNF signaling

Alessandra Martire[1], Fikru Belema Bedada[1], Shizuka Uchida[1], Jochen Pöling[1,3], Marcus Krüger[1], Henning Warnecke [2,3], Manfred Richter[4], Thomas Kubin[1], Susanne Herold[5], Thomas Braun[1]

[1] Max-Planck-Institute for Heart and Lung Research, Department of Cardiac Development and Remodelling, Ludwigstrasse 43, 61231 Bad Nauheim, Germany

[2] Institute for Heart and Circulation Research, University of Witten/Herdecke, Alfred-Herrhausen-Straße 50, 58448 Witten, Germany

[3] Department of Cardiac Surgery, Schüchtermann-Klinik, Ulmenallee 11, 49214 Bad Rothenfelde, Germany

[4] Department of Department of Cardiac Surgery, Kerckhoff-Clinic, Benekestrasse 2-8, Bad Nauheim, 61231, Germany

[5] Universities of Giessen & Marburg Lung Center, Department of Internal Medicine II, Klinikstr.33, 35392 Giessen, Germany, member of the German Center for Lung Research (DZL)

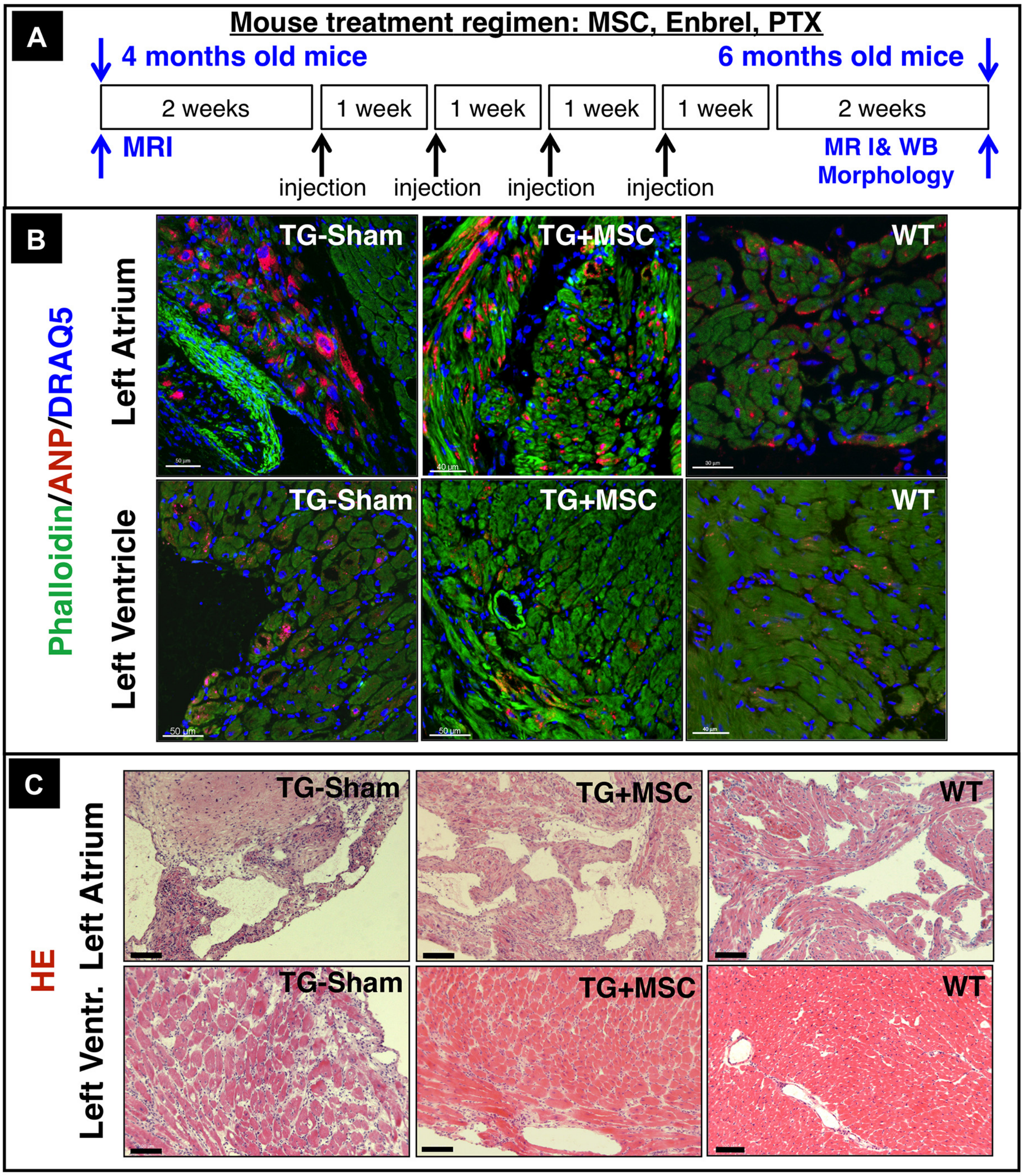

Supplement Fig. S1: Reduced expression of ANP and reduced accumulation of infiltrating mononuclear cells in hearts of αMyHC-MCP-1 mice after administration of MSC. (A) Outline of the treatment regimen consisting of 4 weekly injections of MSCs, Enbrel, or PTX. Mice were examined by MRI at 4 and 6 months of age followed by morphological and western blot analysis. **(B)** ANP immunofluorescence staining (red) of sections from left atriae and ventricles of TG and WT control mice after sham-treatment or MSC injections. Sections were counterstained with phalloidin (green) and DRAQ5 to label cardiomyocytes and nuclei, respectively. Scale bars upper row: 50 μm, 40 μm, 30 μm (from left to right). Scale bars lower row: 50 μm, 50 μm, 40 μm (from left to right). **(C)** Hematoxylin/Eosin staining of sections from left atriae and ventricles of TG and WT control mice after sham-treatment or MSC injections. Scale bars: 100 μm.

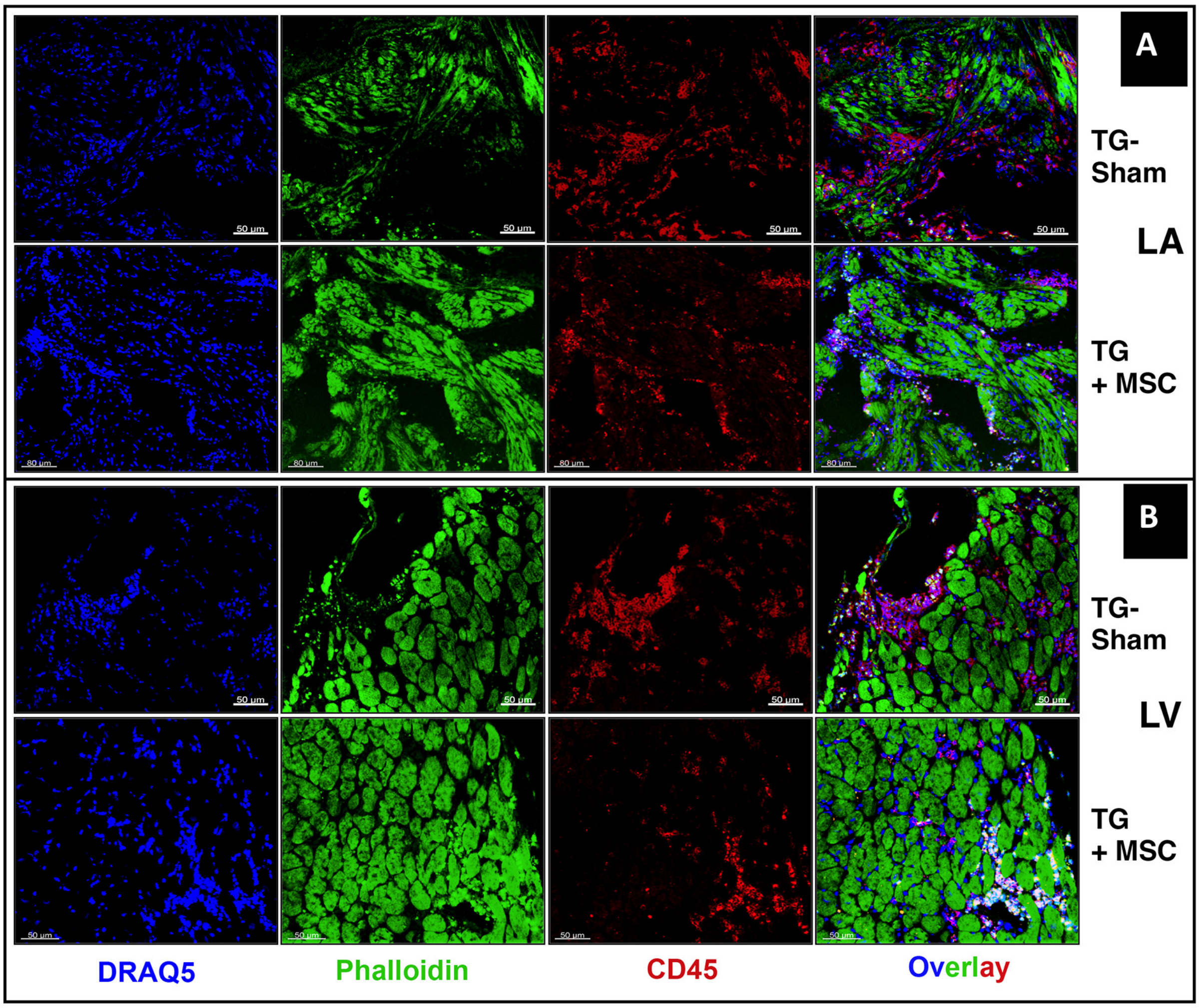

Supplement Fig. S2: Reduced accumulation of inflammatory cells in hearts of αMyHC-MCP-1 mice after administration of MSC. (A, B) Confocal images of CD45 (red) stained sections from left atriae (A) and ventricles (B) of αMyHC-MCP-1 mice (TG) after sham-treatment or intravenous injection of MSC. Sections were counterstained with phalloidin (green) and DRAQ5 (blue) to label cardiomyocytes and nuclei, respectively. Scale bars: 50 μm.

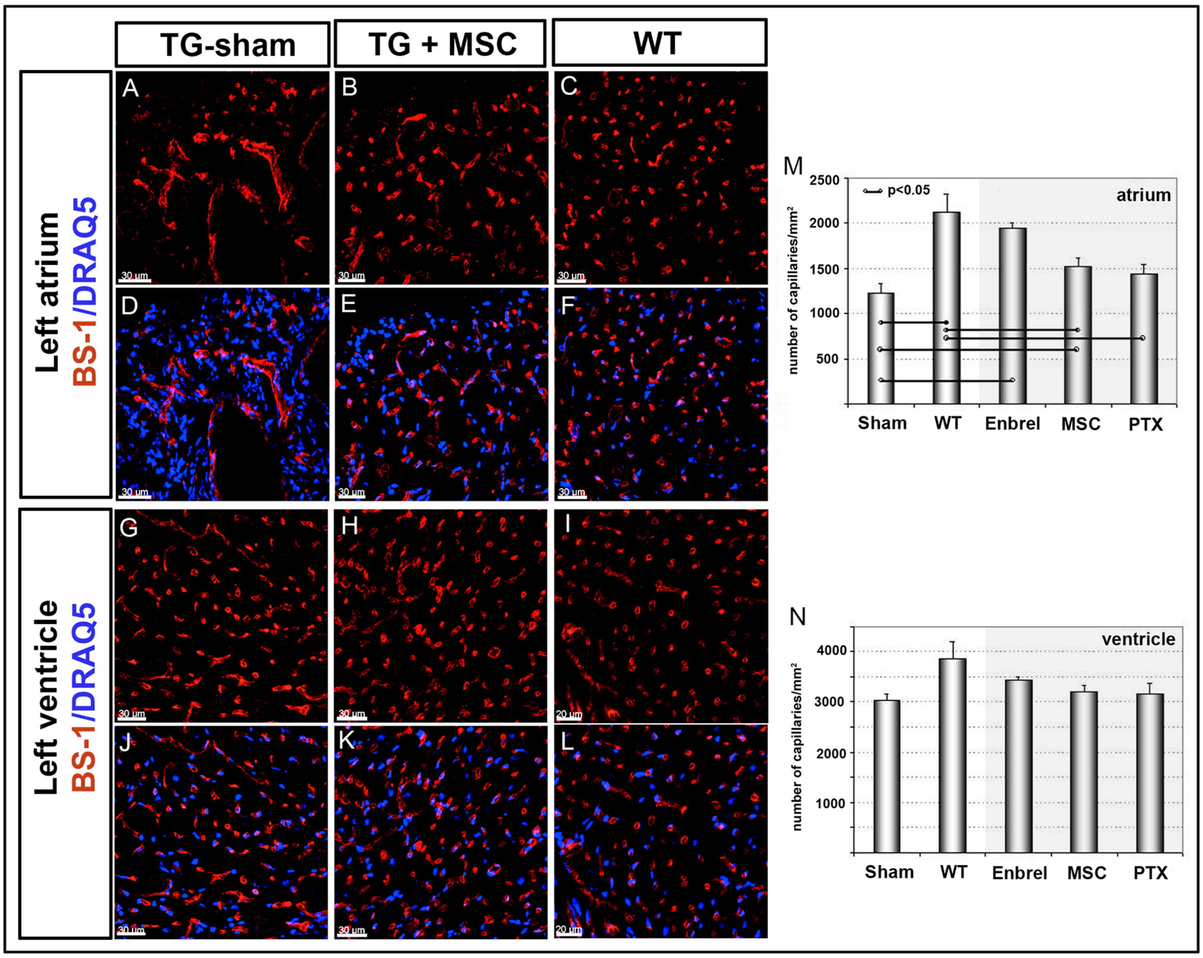

Supplement Fig. S3: Increased capillary density in the left atrium of MSC or Enbrel treated αMyHC-MCP-1 mice. (A-L) BS-1 (endothelial cell marker) immunofluorescence staining (red) of sections from left atriae and ventricles of TG and WT control mice, which received sham-treatment, drug treatment or MSC injections as indicated. Sections were counterstained with DRAQ5 (blue) to label nuclei. **(M, N)** Statistical evaluation of increased capillary density in the left atrium (M) but not the left ventricle (N) of treated αMyHC-MCP-1 (TG) mice at 6 months of age. PTX: Pentoxifiline. Scale bars: (A-H, J, K): 30 µm; (I, L): 20 µm.

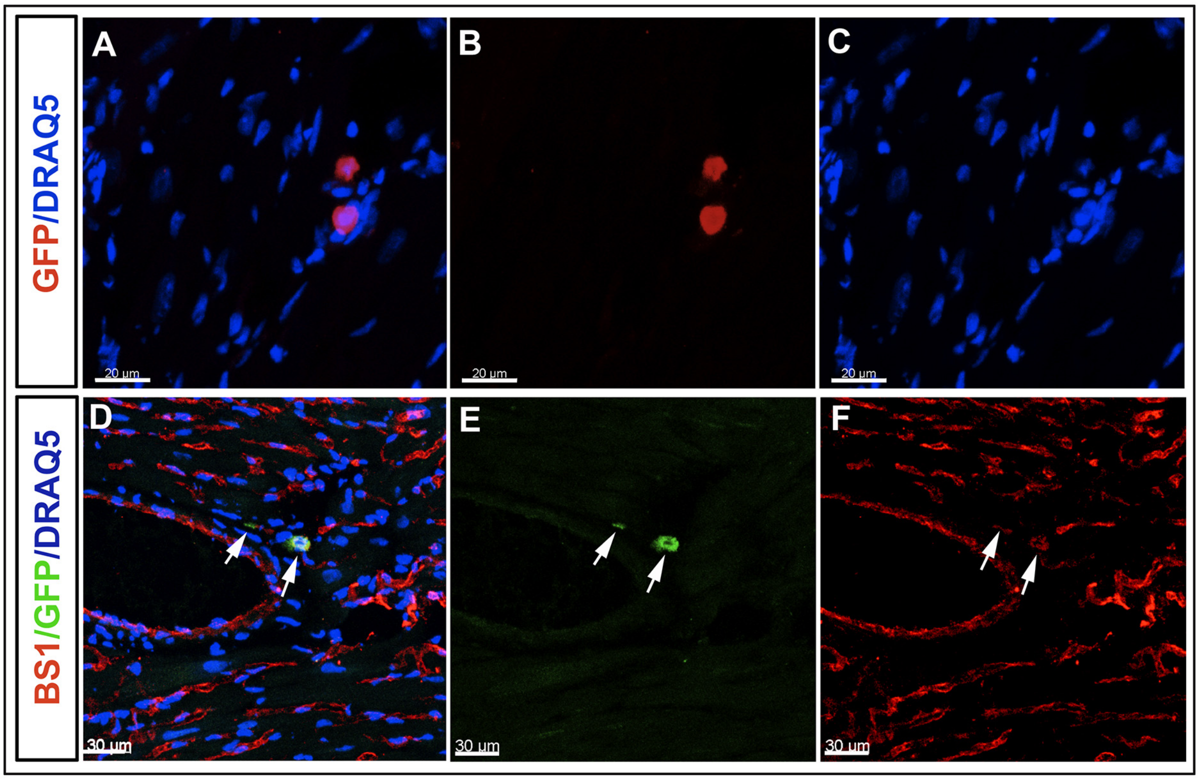

Supplement Fig. S4: Limited contribution of MSC to the endothelial lineage after homing of into hearts of αMyHC-MCP-1 mice. (A-F) Immunofluorescence images of sections through the left ventricle (LV) of αMyHC-MCP-1 mice after intravenous administration of GFP-labeled MSC. **(A-C)** Few GFP-labeled MSC (red) remain in the ventricular myocardium 3 weeks after the last injection of MSC. **(D-F)** Some of the remaining MSC (green) express the endothelial cell marker BS-1 (red). Double-positive cells are marked by arrows. Sections were counter-stained with DRAQ5 to label nuclei. Scale bars: (A-C): 20 µm; (D-F): 30 µm.

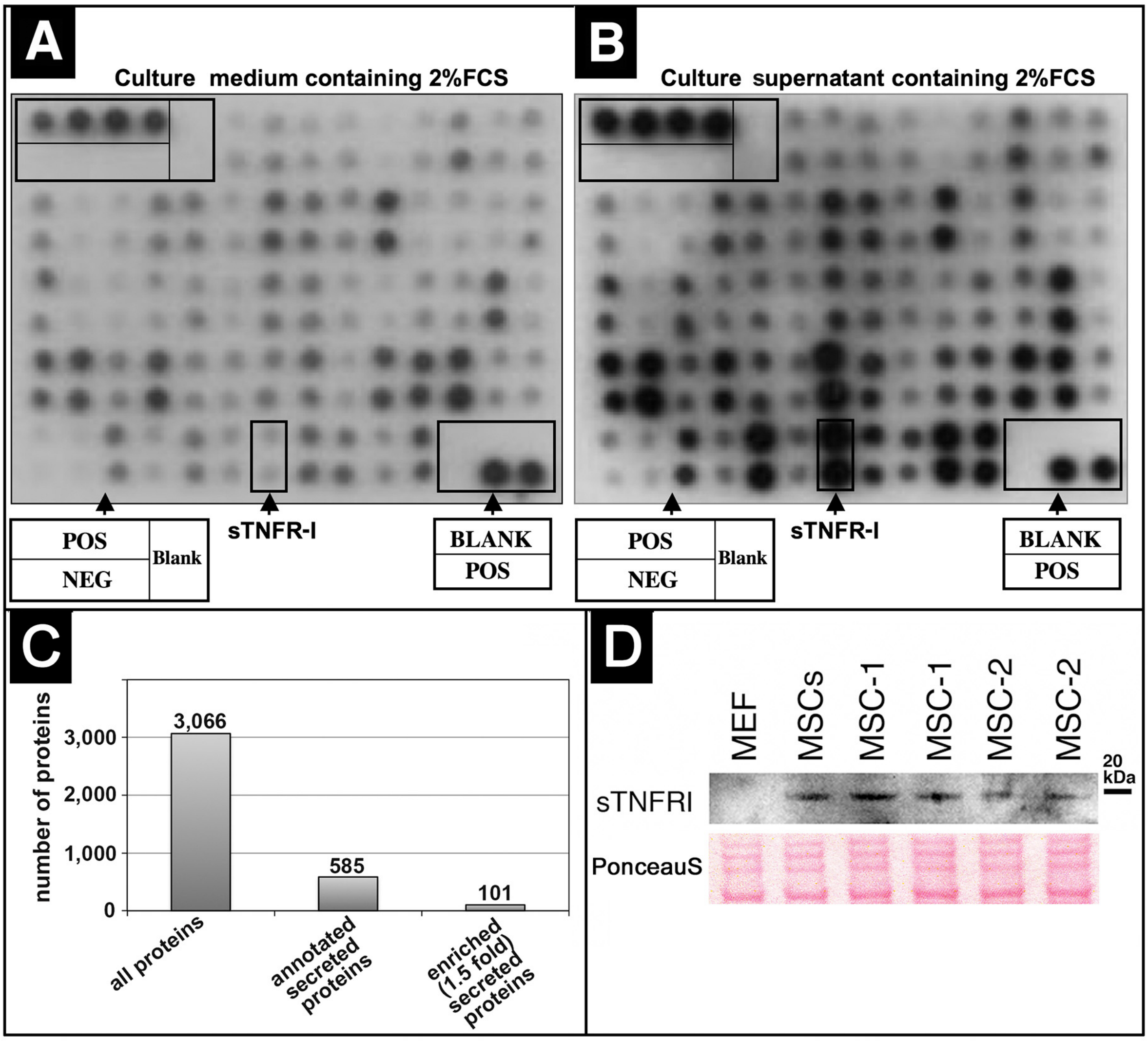

Supplement Fig. S5: MSC release numerous proteins including sTNF-RI. (A, B) Cytokine array (mouse cytokine array 3.1.; RayBiotech) probed with culture medium containing 2% FCS as a control (A) and with 2% FCS plus culture supernatant from MSC (B). The two spots corresponding to sTNF-RI are boxed. Positive (POS) and negative controls (NEG) and blanks (BLANKS) are indicated. **(C)** SILAC-assisted mass spectrometry and protein array analysis led to the identification of 3,066 proteins in the supernatant of cultured MSCs. GO-annotation analysis identified 585 proteins, which are known to be secreted, localized at the plasma membrane, or present in the cytosol. 101 out of the 585 putatively secreted proteins were found at higher levels in supernatants of MSC compared to 10T1/2 cells as determined by quantitative SILAC mass spectrometry. **(D)** Western blot analysis of sTNF-RI in the supernatants of mouse embryonic fibroblasts (MEF), two clones of MSC (MSC-1, MSC-2) and primary MSC preparations (MSC). Loading was normalized to Ponceau S staining of the membrane.

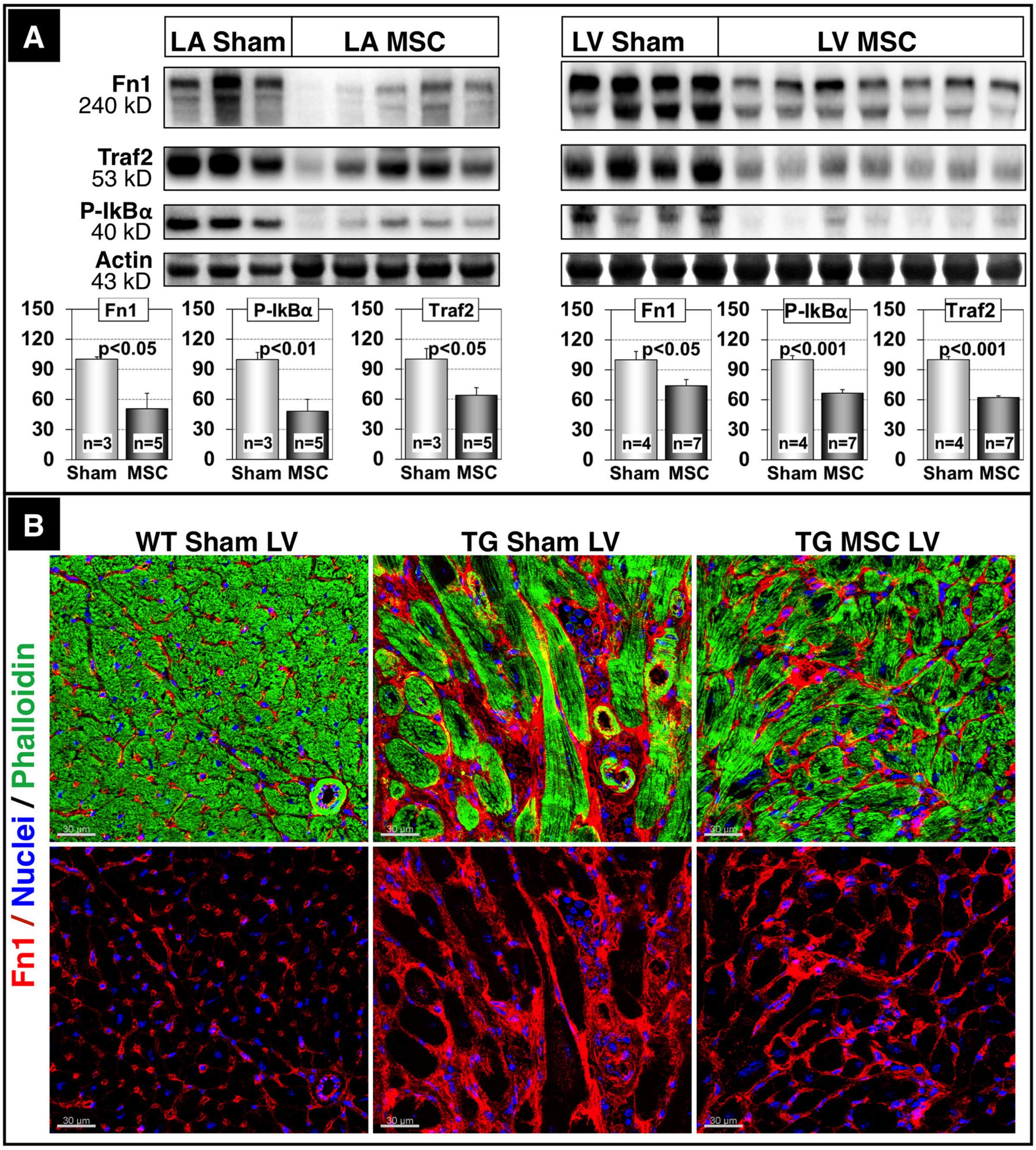

Supplement Fig. S6: Administration of MSC reduces expression of p65 targets genes and increases phosphorylation of IκB in hearts of αMyHC-MCP-1 (TG) mice. (**A**) Western blot analysis of the concentration of fibronectin (Fn1), phosphorylated-IkBα (Ser32; P-IκBα) and Traf2 in left atriae (LA) and left ventricles (LV) of 6-months old wild-type (WT), αMyHC-MCP-1 (TG) and MSC-treated αMyHC-MCP-1 (TG) mice. (**B**) Immunofluorescence analysis of expression of the p65 target gene fibronectin (Fn1) in the ventricular myocardium of 6-months old wild-type (WT), αMyHC-MCP-1 (TG) and MSC-treated αMyHC-MCP-1 (TG) mice.

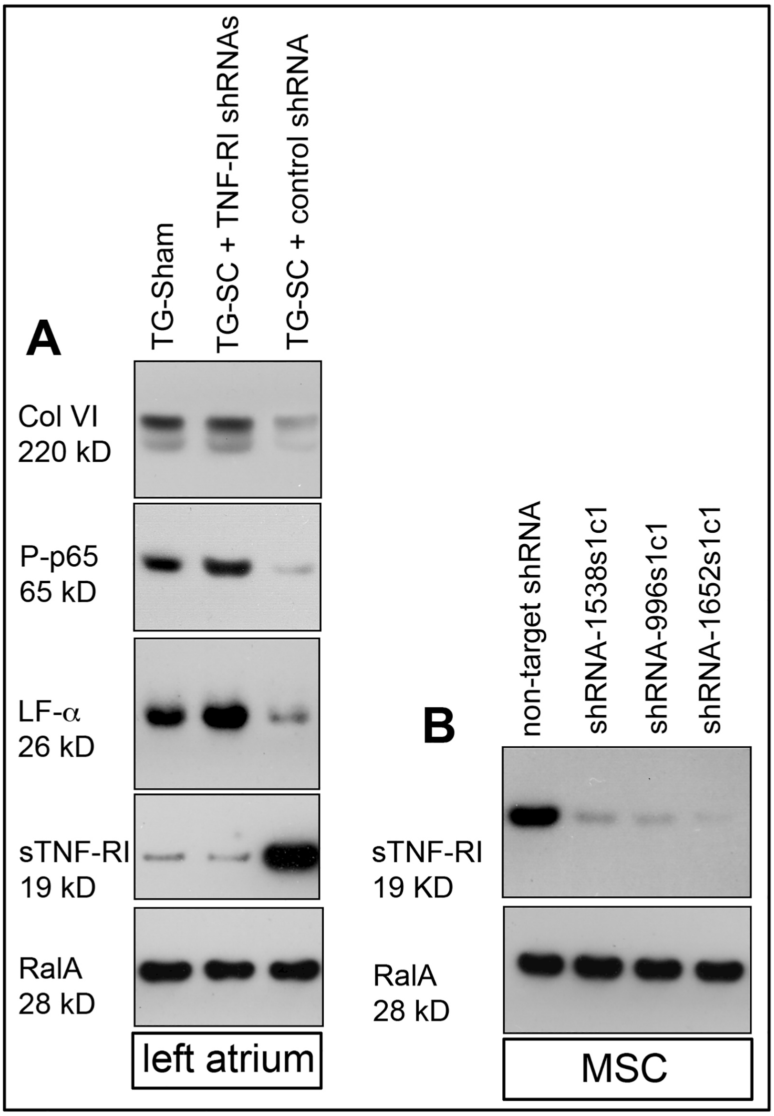

Supplement Fig. S7: shRNA mediated knock-down of TNF-RI abrogates positive effects of MSC in the left atrium (LA) of αMyHC-MCP-1 mice. (A) Western blot analyses of tissue lysates from the left atrium of 6-months old αMyHC-MCP-1 mice (TG) after sham-treatment and intravenous injection of MSC. MSC were infected with lentiviruses coding for a control shRNA or a shRNA directed against sTNF-RI before injection into TG mice. (B) Western blot analysis of the culture supernatant of MSC after infection with lentiviruses carrying a non-target control shRNA or three different shRNAs directed against TNF-RI. All three shRNAs efficiently suppressed secretion of sTNF-RI. RalA was used as a loading control.

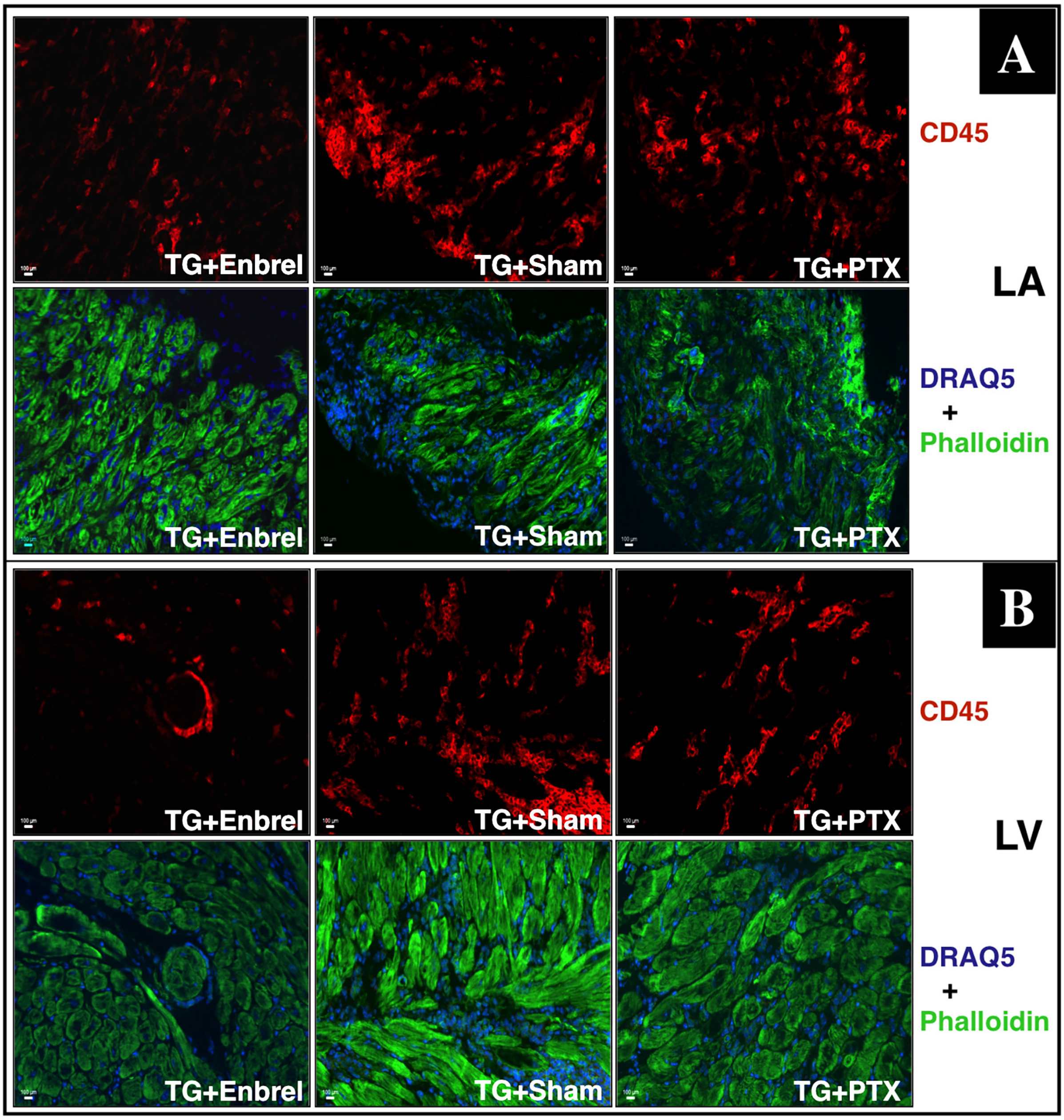

Supplement Fig. S8: Reduced accumulation of inflammatory cells in hearts of αMyHC-MCP-1 mice after treatment with Enbrel or PTX. (A-B) Confocal images of CD45 (red) stained sections from left atriae (LA) and ventricles (LV) of 6 month old αMyHC-MCP-1 mice (TG) after sham-treatment or administration of Enbrel or PTX. Sections were counterstained with phalloidin (green) and DRAQ5 (blue) to label cardiomyocytes and nuclei, respectively. Scale bars: 100 μm.
